# High-risk human papillomavirus clearance in pregnant women: trends for lower clearance during pregnancy with a catch-up postpartum

**DOI:** 10.1038/sj.bjc.6600367

**Published:** 2002-07-15

**Authors:** M A E Nobbenhuis, T J M Helmerhorst, A J C van den Brule, L Rozendaal, P D Bezemer, F J Voorhorst, C J L M Meijer

**Affiliations:** Department of Pathology, Vrije Universiteit Medical Center, PO Box 7057, 1007 MB, Amsterdam, The Netherlands; Department of Obstetrics and Gynaecology, University Hospital Rotterdam, PO Box 2040, 3000 CA, Rotterdam, The Netherlands; Department of Clinical Epidemiology and Biostatistics, Vrije Universiteit Medical Center, PO Box 7057, 1007 MB, Amsterdam, The Netherlands

**Keywords:** human papillomavirus, pregnancy, follow-up study, natural history, prevalence

## Abstract

We followed 353 women referred with abnormal cervical cytology in a non-intervention cohort study. In 91 pregnant women we compared high-risk human papilloma virus rates in the subsequent trimesters and postpartum in comparison to 262 non-pregnant women. High-risk human papilloma virus clearance was compared with 179 high-risk human papilloma virus positive non-pregnant women. Our main questions were: (1) do high-risk human papilloma virus rates change during pregnancy?; and (2) is there any difference between high-risk human papilloma virus clearance in pregnant and non-pregnant women? Women were monitored 3–4 monthly by cytology, colposcopy, and high-risk human papilloma virus testing. The median follow-up time was 33 months (range 3–74). Non-pregnant women showed prevalence rates of high-risk human papilloma virus of 64, 57, 53, and 50%, respectively, in four subsequent 3-months periods since the start of the study. These high-risk human papilloma virus rates were higher than in the three trimesters of pregnancy, and during the first 3 months postpartum, i.e. 50, 44, 45, and 31%, respectively. Postpartum only, this difference was statistically significant (*P*=0.004). Paired comparisons of high-risk human papilloma virus prevalence rates of the different trimesters with the postpartum rate showed (McNemar test) decreased rates: first trimester: 18% (*P*=0.02), second trimester: 13% (*P*=0.02) and third trimester: 23% (*P*<0.005). Such a phenomenon was not found in non-pregnant women. Pregnant women showed a trend for increased high-risk human papilloma virus clearance during the third trimester and postpartum compared to non-pregnant women (hazard ratios 3.3 (0.8–13.7) and 4.6 (1.6–12.8), respectively). These results suggest a lowered immune-response against human papilloma virus during the first two trimesters of pregnancy with a catch-up postpartum.

*British Journal of Cancer* (2002) **87**, 75–80. doi:10.1038/sj.bjc.6600367 www.bjcancer.com

© 2002 Cancer Research UK

## 

Several epidemiological and biological studies have established the important role of infection with high-risk types of human papilloma virus (HPV) for development of cervical cancer and its precursor lesions. In women with or without abnormal cervical smears a positive high-risk HPV test result indicates an increased risk for development of high grade cervical lesions (Ho *et al*, 1995; Remmink *et al*, 1995; Rozendaal *et al*, 1996, 2000; Nobbenhuis *et al*, 1999). Moreover, in nearly all cervical cancers high-risk HPV types have been detected (Walboomers *et al*, 1999).

The increased prevalence of high grade cervical lesions and cervical cancer in immunocompromised patients, such as AIDS patients and transplant recipients, indicates that persistence of high-risk HPV and consequently HPV-mediated carcinogenesis is related to compromised immunosurveillance (IARC, 1995; Petry *et al*, 1996; Sun *et al*, 1997). Pregnancy is believed to alter immune-response in women (Schneider *et al*, 1987; Sethi *et al*, 1998). Some authors concluded pregnancy has no effect on CIN (Jain *et al*, 1997). Others reported high regression rates of cervical dysplasia in the postpartum period (Yost *et al*, 1999). In contrast, high prevalence rates of high-risk HPV have been found in pregnant women (Rando *et al*, 1989; Saito *et al*, 1995; Morrison *et al*, 1996; Fife *et al*, 1999), although in other studies no differences in HPV prevalence between pregnant and non-pregnant women were seen (Peng *et al*, 1990; Kemp *et al*, 1992; De Roda Husman *et al*, 1995a). In short, the influence of pregnancy on the natural course of infection with high-risk HPV types is not yet known.

We performed a non-intervention follow-up study in 353 women referred for colposcopy because of abnormal cervical cytology and compared high-risk HPV clearance in pregnant women with that of non-pregnant women. Our main questions were: (1) do high-risk HPV rates change during pregnancy?; and (2) is there any difference between HPV clearance in pregnant and non-pregnant women?

## MATERIALS AND METHODS

### Women

From June 1990 to December 1992, 353 women referred to the colposcopy clinic of the University Hospital Vrije Universiteit, Amsterdam, were followed in a non-intervention study. The characteristics of this cohort have been described previously (Nobbenhuis *et al*, 1999, 2001). Briefly, the inclusion criteria were: referral because of an abnormal cervical smear (i.e. mild to severe dyskaryosis); age 18–55 years; no history of cervical pathology, prenatal diethylstilbestrol exposure, or concomitant cancer; no HIV or immunosuppression, and sufficient Dutch or English language skills. The median follow-up time was 33 months (range 3–74). Enrolment into the study (baseline) took place about 2 months after referral.

**Figure 1:**
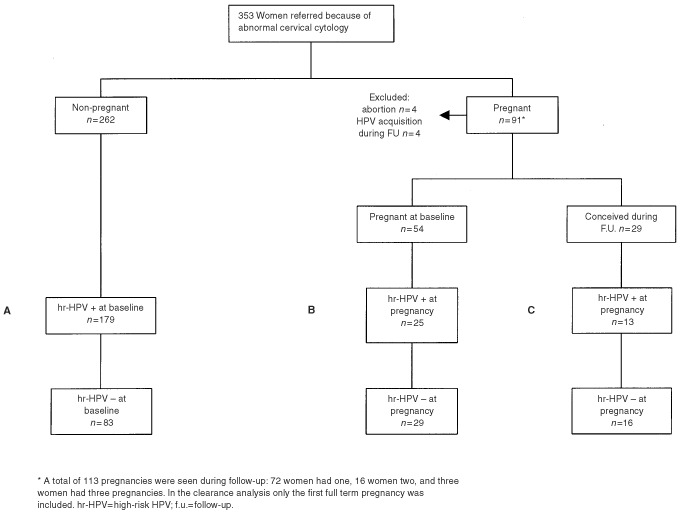
Characteristics of study groups.

Ninety-one women were pregnant during follow-up. The first recorded full term pregnancy in each woman was used to computer-high-risk HPV prevalence rates and analyse high-risk HPV clearance. For the analysis of high-risk HPV clearance we excluded four pregnancies ending in abortion and four women who acquired HPV during follow-up prior to their pregnancy. One hundred and seventy-nine non-pregnant women with a positive high-risk HPV test at baseline (Figure 1

; group A) and 38 pregnant women with a positive high-risk HPV test at their first visit during pregnancy (Figure 1; groups B and C) were used to study high-risk HPV clearance between pregnant and non-pregnant women.

Women were monitored every 3 to 4 months by cytology, colposcopy and HPV testing (Remmink *et al*, 1995; Nobbenhuis *et al*, 1999, 2001). Clinicians and laboratory staff were blinded for HPV test results and clinical findings, respectively. No biopsy samples were taken during follow-up to avoid any interference with the natural course of the disease. Follow-up ended if women reached clinical progression (*n*=33), defined as a colposcopic impression of CIN 3 covering three or more cervical quadrants or a cervical smear suspect for microinvasive carcinoma, or at the end of the study in December 1996. At the last visit, colposcopically directed biopsy samples were taken for histological verification of suspected lesions (=end histology). In women with normal colposcopy, random samples were taken. If necessary, women were treated according to standard protocols.

The study protocol was approved by the ethics review board of the hospital, and all women voluntarily signed informed consent before enrolment.

### Human papillomavirus testing

Testing for HPV was done by EIA PCR, which used HPV general-primer-mediated PCR with the general primers GP 5+/6+ to detect a broad spectrum of mucosotropic HPV types (De Roda Husman *et al*, 1995b; Jacobs *et al*, 1997). PCR products were used to identify in one assay all 14 high-risk types using EIA (HPV type 16, 18, 31, 33, 35, 39, 45, 51, 52, 56, 58, 59, 66 and 68). In addition, individual high-risk HPV types were determined. This test has been described previously and has been clinically validated (Jacobs *et al*, 1997, 2000).

### Definition of HPV clearance

Women were considered to be clear of infection when no high-risk HPV type from the previous visit was detected at the next visit (Nobbenhuis *et al*, 1999, 2001).

### Statistical analysis

We compared pregnant and non-pregnant women in 3 months periods. In non-pregnant women these periods were designated from the start of the study. Age distribution at baseline and at time of pregnancy was compared by the Mann–Whitney test. The Fisher-exact test was used to calculate differences in high-risk HPV rates and abnormal cervical smears in pregnant and non-pregnant women. We also analysed the prevalence of low-risk HPV types and multiple HPV infections at different trimesters but the numbers were too small to draw any conclusions (data not shown).

Not all women were seen in each trimester of pregnancy. Therefore comparisons for cytology and HPV samples between first, second, and third trimester, or postpartum were made by the McNemar test, only for those women with specimens available at both times. Being interested in the effect of pregnancy, only the first postpartum visit within 6 months after delivery was included in the calculation (range 5–25 weeks after pregnancy). The clearance of high-risk HPV in pregnant and non-pregnant women and between pregnancy groups (pregnant at the start of the study versus conceived during the study) were studied by Kaplan–Meier curves during a period of 12 months. For contrasts we used the log-rank test. The time-point of HPV clearance was taken at the mid-point between positive and negative test results for high-risk HPV (Nobbenhuis *et al*, 1999, 2001). We included 179 non-pregnant women with a high-risk HPV positive test at baseline (Figure 1; group A) and 38 women with a high-risk HPV positive test at their first visit during pregnancy (Figure 1; groups B and C). In pregnant women the baseline starting-point for the analysis was the last menstruation. Cox-regression was performed to calculate the relative risks (Hazard Rates) to clear high-risk HPV in pregnant and non-pregnant women in different time periods. Adjustment was made for women who conceived during follow-up. Throughout all analyses, a value of *P*<0.05 was considered significant. We did not correct for multiple testing.

## RESULTS

The median age in the pregnant and non-pregnant group of women was 30 years (range 20–44 years) and 32 years (range 19–55 years), respectively (*P*=0.23). At baseline 68% (179 out of 262) of non-pregnant women had a positive high-risk HPV test result compared to 63% (57 out of 91) of pregnant women (*P*=0.32). At baseline no significant difference in number of abnormal cervical smears was observed in high-risk HPV positive pregnant and non-pregnant women; 133 out of 179 (74%) non-pregnant women still had an abnormal cervical smear compared to 15 out of 25 (60%) pregnant women (*P*=0.13).

Among pregnant women with cervical smears obtained in the first trimester of pregnancy 39% (21 out of 54 available smears) had abnormal cervical cytology (mild dyskaryosis or worse). Abnormal cytology in the second, and third trimester and postpartum was present in 42% (25 out of 60), 33% (13 out of 39), and 34% (27 out of 79 available smears), respectively. Comparison of paired cervical smear samples among the different trimesters with the postpartum visit did not show any significant difference (*P*>0.1). In non-pregnant women no significant difference in abnormal cervical smears among the different time periods was found (data not shown).

### Prevalence rates of high-risk HPV

In non-pregnant women the prevalence rates of high-risk HPV obtained from the available samples at the different time periods (i.e. 3, 6, 9 and 12 months of follow-up) were 64, 57, 53 and 50%, respectively (Table 1

**Table 1:**
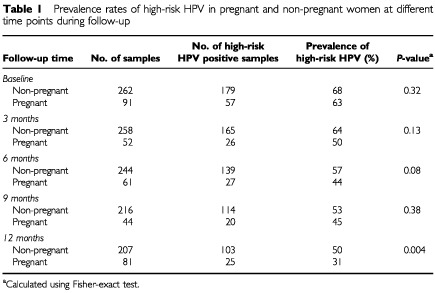
Prevalence rates of high-risk HPV in pregnant and non-pregnant women at different time points during follow-up

).

Among pregnant women whose HPV samples were obtained in the first trimester of pregnancy 50% had positive high-risk HPV DNA samples (Table 1). The high-risk HPV prevalence rates in the second and third trimester, and postpartum were 44, 45 and 31%, respectively. Of the remaining 10 women without a postpartum visit, six women wanted to withdraw from the study and had a cervical biopsy during pregnancy, and four women had a follow-up visit more than 6 months postpartum. High-risk HPV positive rates in three monthly periods in the non-pregnant groups were not significantly higher than during pregnancy trimesters and postpartum (Table 1).

Comparison of paired samples of high-risk HPV among the three trimester groups in the pregnant women group did not show any statistical difference (data not shown). In 45 women with paired samples in the first trimester and postpartum a significant decline in high-risk HPV positive tests of 51% (23 out of 45) to 33% (15 out of 45) was found (*P*=0.02). Comparing second trimester results with postpartum results (paired data available from 53 women) showed a significant decline from 45% (24 out of 53) to 32% (17 out of 53) high-risk HPV positive tests (*P*=0.02). A significant difference in high-risk HPV rates was also found between the third trimester and postpartum (paired data available from 40 women) from 48% (19 out of 40) to 25% (10 out of 40) (*P*=0.004).

### High-risk HPV clearance

Twenty-five high-risk HPV positive women (66%) were pregnant at baseline (Figure 1; group B) and 13 (34%) became pregnant during follow-up (Figure 1; group C). We used both groups to analyse high-risk HPV clearance. To exclude potential bias for combining these two groups in relation to the starting point we analysed the influence of this heterogeneity. Kaplan–Meier curves did not show differences in clearance rates between the two pregnancy groups or with the non-pregnant reference group (log-rank tests: n.s.). Adjustment for pregnancy groups in Cox regression analysis did not significantly effect the computed relative risks. Moreover, we did not find significant differences between the relative risks in the pregnancy groups. Thus, we did not find any evidence that combining both groups introduces bias.

Among 38 pregnant women with a high-risk HPV positive test at the first visit during pregnancy the cumulative 12-months incidence of high-risk HPV clearance was 42% (95% C.I. 25–59; figure not shown). One hundred and seventy-nine non-pregnant women with a positive high-risk HPV test at baseline had a cumulative 12-months incidence of high-risk HPV clearance of 31% (95% C.I. 24–38; figure not shown). Both clearance curves according to Kaplan–Meier analysis cross at 9 months thus excluding long term effects on prevalence rates. The crude trimester dependent hazard ratio's, and the trimester dependent hazard ratio adjusted for pregnancy groups for HR–HPV clearance (Table 2

**Table 2:**
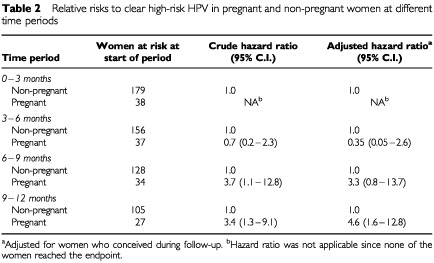
Relative risks to clear high-risk HPV in pregnant and non-pregnant women at different time periods

), show that the differences between the curves are not only related with random variation. At 9 to 12 months of follow-up, i.e. the postpartum period, pregnant women were 4.6 times (95% C.I. 1.6–12.8) more at risk to clear HPV than non-pregnant women followed during the same time since the start of the study. The adjusted hazard ratio's for clearance during the second and third trimester were 0.35 (95% C.I. 0.05–2.6) and 3.3 (95% C.I. 0.8–13.7), respectively. Trends for decreased high-risk HPV clearance in cervical smears in pregnant women in contrast with non-pregnant women are compensated by increased clearance postpartum. The increased clearance probably already starts during the third trimester since the crude hazard ratio was significant during that period. Inclusion of more pregnant women would probably also show a significant adjusted hazard ratio.

## DISCUSSION

Our results show that during pregnancy in a group of women initially referred for colposcopy with an abnormal cervical smear the prevalence of high-risk HPV is higher than in the postpartum period. This effect is also demonstrated by the trimester dependent HPV clearance rates; during the postpartum period women were more at risk to clear high-risk HPV than non-pregnant women.

The high-risk HPV prevalence rates in pregnant women varied between 45 and 50% during the trimesters, and 31% in the postpartum period. Similar figures were found by others. Kemp *et al* (1992) found HPV prevalence rates of up to 42% and Fife *et al* (1999) up to 36% during pregnancy. However, most of these women had normal cervical cytology smears and were performed in a pregnant population of young women with a high incidence of sexual transmitted diseases.

Using a variety of HPV detection techniques, several other studies also have found a significantly higher prevalence of high-risk HPV during pregnancy in comparison with the postpartum period (Rando *et al*, 1989; Saito *et al*, 1995; Fife *et al*, 1999; Yost *et al*, 1999). Several reasons for this phenomenon have been postulated. It has been suggested that pregnant women tend to have fewer sexual partners than non-pregnant women of the same age (De Roda Husman *et al*, 1995a) and therefore have a decreased risk to acquire a new HPV infection (Peng *et al*, 1990; Tenti *et al*, 1997). Pregnancy-related explanations for this change in HPV prevalence have been given in terms of possible selective activation of viruses by hormonal (von Knebel Doeberitz *et al*, 1991; Michelin *et al*, 1997) and immunological factors (Sethi *et al*, 1998). Sethi *et al* (1998) showed that serologic response to HPV type 16 was higher in non-pregnant than in pregnant women suggesting that pregnancy reduces humoral immune response against HPV. In pregnant women we found reduced high-risk HPV clearance during pregnancy with a catch-up postpartum, suggesting that an altered immune-response may be present during pregnancy. Unfortunately, we could not test this since in only a few women blood serum was taken during follow-up. Our study shows that postpartum pregnant women clear HR–HPV faster than non-pregnant women after a same period of follow-up. This downward trend in high-risk HPV prevalence postpartum may be explained by cervical trauma occurring at the time of delivery with additional repair of the cervical epithelium (Yost *et al*, 1999). Indeed, in a study in which postpartum regression rates of cervical lesions were compared in women who delivered vaginally or by caesarean section, a higher postpartum regression was seen in women with vaginal deliveries probably due to stimulation of local immune factors (Ahdoot *et al*, 1998).

From recent studies we know that HPV clearance is associated with and precedes on average 3 months regression of cervical lesions (Nobbenhuis *et al*, 2001; Zielinski *et al*, 2001). Thus, following clearance an increased regression rate of cervical lesions could be expected postpartum. This phenomenon was already described by Yost *et al* (1999). However, during a 12-month period they found no difference in clearance rates between pregnant and non-pregnant women. These results indicate that pregnant women with a high-risk HPV positive test during pregnancy are not at higher risk for progression of underlying lesions than non-pregnant women. Indeed, when we followed our women for an additional 6 months period the cumulative incidence of HPV clearance in pregnant and non-pregnant women became similar (data not shown). In addition, no difference in number of women with progressive CIN was seen in the follow-up of pregnant and non-pregnant women with high-risk HPV positive tests, suggesting that both groups are at similar risks over a period of 1½ years (data not shown).

In conclusion, although at long term the course of a high-risk HPV infection is not affected we found evidence that pregnancy influences high-risk HPV clearance. Trends for a decreased high-risk HPV clearance in the first two trimesters in pregnant women in contrast with non-pregnant women are compensated by increased clearance demonstrable postpartum but already starting in the third trimester.
